# Development of a brief, generic, modular resource-use measure (ModRUM): piloting with patients

**DOI:** 10.1186/s12913-023-10011-x

**Published:** 2023-09-15

**Authors:** Kirsty Garfield, Joanna C. Thorn, Sian Noble, Samantha Husbands, Will Hollingworth

**Affiliations:** 1 Health Economics Bristol, Population Health Sciences, Bristol Medical School, University of Bristol, 1-5 Whitladies Road, Bristol, BS8 1NU UK; 2 Bristol Trials Centre, Population Health Sciences, Bristol Medical School, University of Bristol, 1-5 Whiteladies Road, Bristol, BS8 1NU UK

**Keywords:** Resource-use measurement, Self-report, Questionnaire development, Questionnaire validation

## Abstract

**Background:**

Bespoke self-report resource-use measures (RUMs) are commonly developed or adapted for each new randomised controlled trial. Consequently, RUMs lack standardisation and validation is rarely conducted. A new generic RUM, ModRUM, has been developed using a rigorous process, including consultation with health economists and patients. ModRUM includes a concise core healthcare module, designed to be included in all trials, and depth-adding questions, which can replace or be added to core questions as needed. Modules covering other sectors are under development. The aim of this study was to test the acceptability, feasibility, and criterion and construct validity of the healthcare module of ModRUM.

**Methods:**

Patients who had a recent appointment at their GP practice were invited to complete ModRUM (core module or core module with depth questions), a characteristics form and the EQ-5D-5L. Acceptability was assessed via response rates and questionnaire completion time. Feasibility was assessed by reviewing issues observed in participants’ responses and question completion rates. Construct validity was tested via hypothesis testing and known-group analyses, using Wilcoxon rank-sum and Kruskal–Wallis tests, and a generalised linear model. Criterion validity was tested by comparing ModRUM results with primary care medical records. Sensitivity, specificity, and agreement using Lin’s concordance correlation coefficient (p_c_) were estimated.

**Results:**

One hundred patients participated from five GP practices in the South-West of England. Acceptability was higher for the core module (20% versus 10% response rate). Question completion rates were high across both versions (> 90%). Some support was observed for construct validity, with results suggesting that healthcare costs differ dependent on the number of long-term conditions (*p* < 0.05) and are negatively associated with health-related quality of life (*p* < 0.01). Sensitivity was high for all questions (> 0.83), while specificity varied (0.33–0.88). There was a good level of agreement for GP contacts and costs, and prescribed medication costs (p_c_ > 0.6).

**Conclusion:**

This study provided preliminary evidence of the acceptability, feasibility, and criterion and construct validity of ModRUM. Further testing is required within trials and with groups that were less well represented in this study.

**Supplementary Information:**

The online version contains supplementary material available at 10.1186/s12913-023-10011-x.

## Introduction

Within trial-based economic evaluations, participants are often required to self-report their use of resources in resource-use measures (RUMs). Self-report is a pragmatic approach that allows a wide range of resource-use data to be collected relatively quickly and cheaply [1, 2]. Despite self-report RUMs being a popular approach, there is currently no standardised generic RUM that is relevant and well-utilised in a wide range of trials [2, 3]. Instead, for each new trial, researchers tend to adapt existing or design bespoke RUMs [3]. This leads to a lack of standardisation, which inhibits the comparability of results across trials, which is important when a primary objective of economic evaluation is to inform resource allocation decisions [4, 5]. In addition, within the scope of a trial, it is unlikely that the measurement properties of a bespoke or adapted RUM, including validity and acceptability, will be assessed prior to administration with trial participants.

Recently, a new modular RUM (ModRUM) has been developed, which is designed for use in a wide range of trials. ModRUM includes a core healthcare module, to be collected in all trials, with optional depth questions that can replace or be added to core questions when more detailed information is required for increased precision in cost estimates or when broader healthcare items are relevant (e.g. paramedic care). The items included in the healthcare module were informed by a Delphi consensus study with health economists, where they identified ten core items that should be collected in all trial-based economic evaluations [4]. The face and content validity of ModRUM were then assessed in qualitative interviews with health economists. Once the content of ModRUM was deemed valid by health economists, the acceptability and content validity of ModRUM were assessed in qualitative ‘think-aloud’ interviews with patients recruited from primary care [6]. ModRUM was revised based on findings to improve comprehensibility [6]. ModRUM can be adapted so that it is relevant to a range of trials of different health conditions (e.g. examples can be changed, and/or items pertinent to the trial population can be added). The feasibility of adapting ModRUM was tested by health economists who adapted ModRUM to hypothetically use it for a recently funded trial.

Once the content and face validity of an instrument have been assessed, the remaining measurement properties, including feasibility, acceptability, construct validity and criterion validity, can be tested in a larger quantitative study [7]. The feasibility of a new instrument requires the instrument to be viable for patients to complete and for researchers to administer and analyse [8], while the acceptability assesses whether the instrument is well-received by patients [9]. Construct validity can be established through hypothesis testing to assess whether the association between scores from the instrument correlate as expected to another instrument measuring the same or a related construct, or to a patient characteristic which is hypothesised to be associated with the construct of interest [8]. To test the criterion validity of a new instrument, the scores from the new instrument are compared with the scores of another measure, which is ideally the ‘gold-standard’ measure [8].

This paper reports on a study where a paper-based version of ModRUM was piloted with patients recruited from primary care. The aims of this study were to assess the feasibility, acceptability, and construct and criterion validity of ModRUM.

## Methods

### Data collection

GP practices based in the Bristol, North Somerset or South Gloucestershire regions of England were invited to participate in this study. GP practices were selected to represent a range of deprivation scores and areas. GP practice staff identified, screened and sent postal invitations to eligible patients. Patients were eligible to take part if they were aged 18 or over, capable of understanding and completing a questionnaire in English, capable of giving informed consent, and they had had an appointment (face-to-face or remote) with a member of the clinical team (e.g. GP, nurse) within the previous four weeks.

**Fig. 1:**
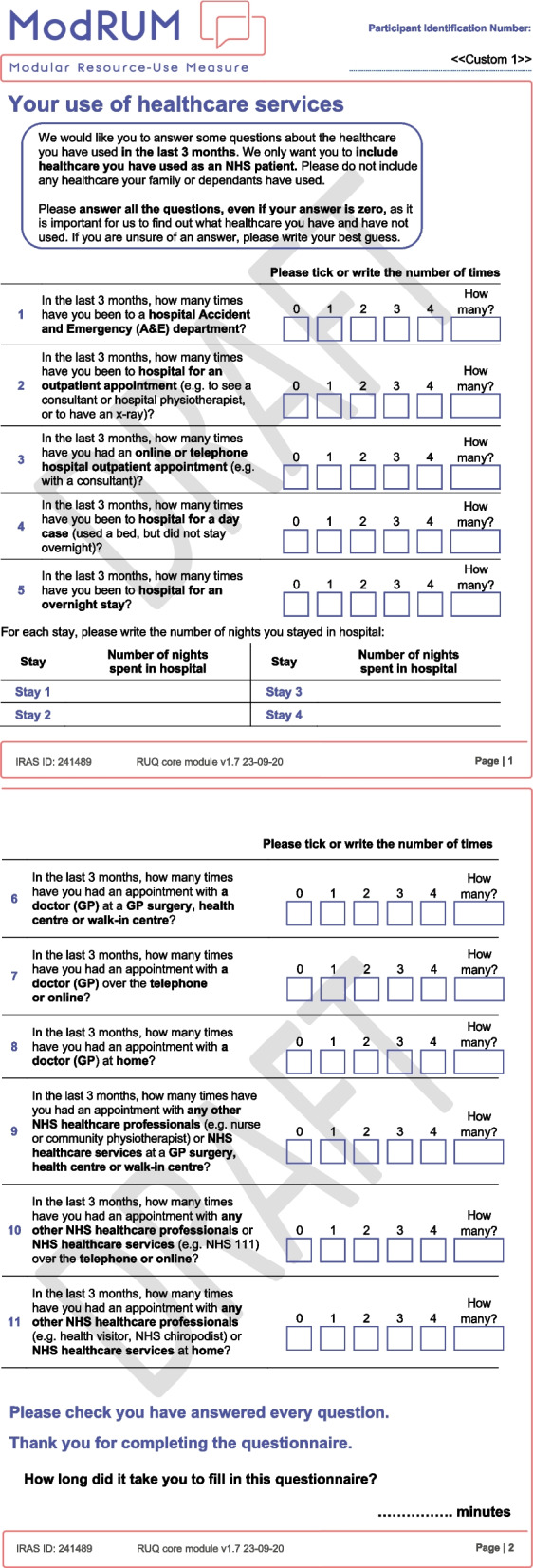
ModRUM core module for piloting with patients

GP practices were assigned to either send ModRUM core module (labelled ModRUM-C, hereinafter) (Fig. 1), or ModRUM core with depth questions (labelled ModRUM-CD, hereinafter) (Additional file 1: Figure S1). All questions referred to a three-month recall period, which represents a commonly used recall period in trials [10]. The target was 800 invitations. To account for potentially lower response rates, more invitations were sent from practices sending ModRUM-CD (*n* = 450) and from practices that were rated as more deprived (*n* = 520) [11]. Patients were also asked to complete the EQ-5D-5L and a patient characteristics form, and to self-report how long it took them to complete ModRUM [12]. The EQ-5D-5L was collected to assess construct validity and increase external validity of the study, as the EQ-5D-5L is commonly completed alongside RUMs in RCTs due to it being the preferred health-related quality of life measure in adults by the National Institute of Health and Care Excellence [13]. Patients who wished to participate were asked to complete and return the documents, and a consent form, in a pre-paid return envelope. At least eight weeks following completion of ModRUM, data on primary care consultations and prescribed medications were extracted from primary care medical records by GP practice staff.

### Data analysis

All analyses were conducted in Stata 17 [14]. Self-reported and medical record resource-use data were cleaned and costed using appropriate national unit costs (Additional file 1: Table S1) [15, 16]. All unit costs were for the year 2019. Where unit costs were not available for 2019, past costs were inflated to 2019 prices using the NHS cost inflation index [15]. Utility values were estimated from EQ-5D-5L scores using a validated mapping function from the EQ-5D-3L [17, 18].

Participant acceptability was assessed using questionnaire response rates and participant-reported completion time. The impact of GP practice deprivation level and ModRUM version on the response rate was also considered using logistic regression. Participant feasibility was assessed using question completion rates and by reviewing issues participants experienced in answering ModRUM questions.

Construct validity was assessed via hypothesis testing including known-group analyses [8, 19]. The following hypotheses were tested: older participants have higher total healthcare costs than younger participants [20], participants with more long-term conditions have higher total healthcare costs than participants with no or one long-term condition [20, 21] and participants with lower self-reported quality-of-life have higher total healthcare costs than those with higher self-reported quality-of-life [22]. Potential associations were also explored for sex, age on leaving full time education and GP practice deprivation level. Known-group validity was assessed using Wilcoxon rank-sum and Kruskal–Wallis H tests [23]. As ModRUM version was not controlled for within these tests, total cost estimates were based on the core questions, which were asked of all respondents. A generalised linear model (GLM), with identity link function and gamma distribution to account for the positively skewed distribution of costs, was employed to assess the relationship between quality-of-life scores and healthcare costs. In the model, a clustered sandwich estimator was used to obtain robust variance estimates that adjust for potential similarity of participants within GP practices. Explanatory variables included ModRUM version, sex, age and GP practice deprivation score. Multiple model specifications were considered and compared using *linktest*, histograms, percentile plots of deviance residuals and Akaike’s information criterion. To assess the correlation between explanatory variables, the variance inflation factor (VIF) was estimated for explanatory variables and a correlation matrix was formed.

Criterion validity was assessed via sensitivity, specificity, Lin’s concordance correlation coefficient (CCC) and Bland–Altman plots [24–26]. For resource use, sensitivity is the proportion of participants that report use of a resource in their medical records, that are correctly identified as using the resource in ModRUM [23]. Specificity is the proportion of participants who have no recorded use of a resource in their medical records, that are correctly identified as not using the resource in ModRUM [23]. Lin’s CCC can be used to compare continuous, non-normally distributed data [24]. It incorporates measures of precision (Pearson’s correlation) and accuracy and is scaled between -1 (perfect reversed agreement) and 1 (perfect agreement) [24]. Following previous studies assessing agreement between self-report and medical record data, Lin’s CCC (*p*_*c*_) was interpreted according to the following categories: poor (less than 0.40), fair (0.40 to 0.59), good (0.60 to 0.74) and excellent (0.75 to 1.00) [27–29].

## Results

Five GP practices took part in this study. Participant-reported data were collected between November 2020 and March 2021, and GP medical record data were obtained between May and June 2021. 717 patients were invited to participate, including 449 invites to complete ModRUM-CD, and 438 invites sent to patients registered at practices in the five deciles of deprivation considered most deprived.

### Acceptability

The response rate was higher for patients invited to complete ModRUM-C (53 participants, 20% response rate) than ModRUM-CD (47 participants, 10% response rate). After controlling for practice deprivation score, for patients who received ModRUM-C, the odds of taking part were 1.74 times as large as for patients who received ModRUM-CD (95% CI: 1.12 to 2.72, *p* = 0.014). After controlling for ModRUM version, a one-unit improvement in the deprivation level of the GP practice, meant that the odds of patients participating increased by a factor of 1.11 (95% CI: 1.04 to 1.19, *p* = 0.003).

Mean and median participant-reported ModRUM completion times were similar for both versions (Additional file 1: Table S2). The maximum reported completion time of 25 min was reported for ModRUM-C, which possibly indicates that some participants included time spent completing all documents in the mail pack. All other times reported were 12 min or less. Once the outlier was omitted, the mean completion time for ModRUM-C reduced to 4.9 min, compared with 5.7 min for ModRUM-CD; however, this did not alter the median time, which was 5 min for both versions of ModRUM.

### Participant characteristics, quality of life and resource use

Participant characteristics are presented in Additional file 1: Table S3. Most participants were of white ethnicity (95%), 63% had at least one long term condition, 61% were female, and 55% were aged 66 or over. The mean EQ-5D-5L utility score for all participants was 0.750 (SD: 0.249) (Additional file 1: Table S4). On average, the utility score was slightly higher for participants who completed ModRUM-C, than for participants who completed ModRUM-CD (0.772 [SD: 0.212] versus 0.726 [SD: 0.285]).

Mean healthcare utilisation and costs are presented in Additional file 1: Table S5. In both versions of ModRUM, remote consultations with a GP were the most commonly used resource (ModRUM-C: 1.9 contacts, ModRUM-CD: 1.8 contacts). For most resources, the mean number of contacts was similar across ModRUM versions, with the exception of GP surgery contacts which was higher for ModRUM-CD (1.18 versus 0.60). Other healthcare professional contacts were higher for ModRUM-C; however, once other healthcare professional and nurse contacts were added for ModRUM-CD, the number of contacts was similar. The mean total cost was higher for ModRUM-CD (£537 (SD: £1045) versus £462 (SD: £802)). The large standard deviation for both versions reflects that a minority of patients had costly inpatient stays.

### Feasibility

The feasibility of answering questions as intended was demonstrated, as minimal data cleaning was required for ModRUM-C. One participant reported only positive answers and the unanswered questions were assumed to be zero. For ModRUM-CD, cleaning involved moving answers that were in the incorrect position to the relevant question (e.g. when GP contacts were reported under other healthcare professional, but the GP question was unanswered).

Prior to cleaning the data, question completion rates ranged from 96 to 100% for ModRUM-C and 91 to 100% for ModRUM-CD. One participant missed an entire page of questions when completing ModRUM-C, while seven participants who completed ModRUM-CD missed at least one page. Of these seven, two participants reported resource use which should have been reported under missed questions (GP/nurse contacts) under the other healthcare professional question, suggesting that they may not have seen the questions, as opposed to missing them intentionally. For ModRUM-CD, five participants did not complete the tick box question, but the answer could often be inferred from answers in the tables. Two participants recorded remote outpatient appointments under the face-to-face outpatient appointment question which preceded it. Five participants either missed the number of times a medication was prescribed, or reported an answer in a different metric to what was asked for.

Several participants who completed ModRUM-CD reported issues with the pre-paid return envelope. The size of the envelope provided was the only option provided by the mailing service; however, given the high-quality paper and additional pages of ModRUM-CD, the study documents only just fitted in the provided pre-paid envelope. Several participants returned ModRUM-CD in their own envelope.

The design of ModRUM means that questions that appear in ModRUM-C are embedded in ModRUM-CD, where for most items, the core question is the top-level question in ModRUM-CD, with a table below to record further details (e.g. clinic type, procedures, length of stay). For participants who completed ModRUM-CD, costs could be estimated using top-level questions only (e.g. number of outpatient appointments) or using more detailed depth questions (e.g. clinic type, tests/procedures performed and reason for outpatient appointment). Estimated costs were higher across most resources when resources were costed using more detailed information (Additional file 1: Table S6). The largest contributors to this difference were hospital inpatient and day case admissions, for which this sample included three participants who had inpatient admissions and three participants who had day case admissions.

### Construct validity

The hypothesis that older patients would have higher total healthcare costs was not supported, as there was no evidence of a difference in total healthcare costs by age group (Table 1). However, in the regression analysis the opposite was observed with under 66-year-olds having higher healthcare costs than over 65 year olds (*p* = 0.002) (Table 2). There was good evidence against the null hypothesis that median total healthcare costs are the same irrespective of number of long-term conditions, which suggests total costs differ dependent on number of long-term conditions (*p* < 0.05). Total healthcare costs as estimated using ModRUM, were also negatively associated with health-related quality of life (*p* < 0.001); in other words, participants with higher self-reported healthcare costs, reported lower EQ-5D-5L scores. Total healthcare costs were positively associated with GP practice deprivation score (*p* < 0.001), with increased healthcare costs observed for participants registered at less deprived GP practices.

**Table 1:** Results from rank tests to assess construct validity, by patient characteristic

**Groups**	**n**	**Rank sum**	***p*-value**
Sex			
Female	54	2379.0	0.521
Male	36	1716.0	
Age group			
18–30	1	29.5	0.538
31–45	14	745.5	
46–55	12	596.5	
56–65	15	624.5	
66–75	26	1251.5	
76 or over	22	847.5	
Number of long-term conditions			
None	36	1299.0	0.013
One	20	821.0	
More than one	30	1621.0	
Age on leaving full time education			
16 or under	37	1597.5	0.618
17 or 18	16	620.0	
19 and over	33	1523.5

**Table 2:** Results from the generalised linear regression analysis to assess construct validity

		**n**	**Adjusted cost (£)**		**Mean difference (95% CI)**		***p*-value**
			**Marginal mean (95% CI)**				
Version	ModRUM-C	47	639	(454 to 823)			
	ModRUM-CD	35	364	(187 to 541)	-275	(-410 to -140)	< 0.001
Sex	Male	34	580	(421 to 739)			
	Female	48	480	(293 to 667)	-100	(-180 to 31)	0.049
Age group	65 and under	40	632	(467 to 797)			
	Over 65	42	416	(220 to 612)	-216	(-350 to -82)	0.002
Ethnic group	Non-white	3	479	(244 to 715)			
	White	79	523	(354 to 692)	44	(-133 to 220)	0.629
Number of long-term conditions	None	36	287	(243 to 331)			
	One	19	348	(227 to 468)	61	(-53 to 175)	0.298
	More than one	27	956	(409 to 1503)	670	(87 to 1,252)	0.024
Age leaving full time education	16 or under	33	477	(337 to 617)			
	17 or 18	16	415	(212 to 619)	-62	(-135 to 12)	0.102
	19 or over	33	617	(424 to 811)	140	(40 to 241)	0.006
EQ-5D-5L score^a^		82			-47	(-57 to -36)	< 0.001
GP practice deprivation score^b^		82			22	(15 to 29)	< 0.001

^a^Rescaled to increments of 0.1

^b^On a scale of 1 to 10, where 1 is most deprived and 10 is least deprived

### Criterion validity

High sensitivity across all resources (> 0.83), indicated that participants were likely to report healthcare use when it was recorded in the medical records that they had used the resource (Table 3). The low specificity score for GP contacts and wide confidence intervals (0.33, (95% CI: 0.10 to 0.65)) were likely impacted by the low proportion of participants who had not had a GP contact. When compared with healthcare professional contacts, specificity for prescribed medications was relatively high at 0.88 (95% CI: 0.47 to 1.00).

**Table 3:** Estimated sensitivity and specificity of ModRUM compared with medical record data

				**Sensitivity (95% CI)**	**Specificity (95% CI)**
**General practitioner contacts**					
		ModRUM			
		Yes	No		
Medical record	Yes	80	2	0.98	0.33
	No	8	4	(0.92 to 1.00)	(0.10 to 0.65)
**Other healthcare professional contacts**					
		ModRUM			
		Yes	No		
Medical record	Yes	80	2	0.84	0.55
	No	8	4	(0.70 to 0.93)	(0.39 to 0.70)
**Prescribed medications**					
		ModRUM			
		Yes	No		
Medical record	Yes	80	2	0.97	0.88
	No	8	4	(0.86 to 1.00)	(0.47 to 1.00)

Mean resource use and costs were higher in ModRUM than the medical records for GP and other healthcare professional contacts (Table 4). For GPs, the mean difference in contacts and costs were 0.4 (95% CI: 0.1 to 0.7) and £16 (95% CI: £5 to £27), respectively. For other healthcare professionals, the mean difference in contacts and costs were 0.6 (95% CI: 0.1 to 1.1) and £24 (95% CI: £8 to £40), respectively. The estimated mean cost of prescribed medications was 42% higher for GP medical record data than ModRUM data. Based on Lin’s CCC, a good level of agreement was observed between ModRUM and medical records for GP contacts and costs, and prescribed medication costs. For other healthcare professional contacts and costs, there was a poor level of agreement. Based on the 95% limits of agreement, a smaller range of differences between data sources for each individual was observed for GP than other health care professional contacts, while for costs, the largest range was for prescribed medications.

**Table 4:** Estimated agreement between ModRUM and medical record healthcare contacts and costs, by healthcare item

	n	ModRUM		Medical records		Mean difference		*p*_*c*_^a^	95% limits of agreement
		Mean	(SD)	Mean	(SD)	(95% CI)			
*General practitioner*									
Contacts	94	2.8	(2.2)	2.4	(2.09)	0.4	(0.1 to 0.7)	0.693	(-2.8 to 3.6)
Cost (£)	94	80	(69)	64	(57)	16	(5 to 27)	0.602	(-92 to 124)
*Other healthcare professionals*									
Contacts	91	1.7	(2.4)	1.1	(1.3)	0.6	(0.1 to 1.1)	0.224	(-4.0 to 5.2)
Cost (£)	91	36	(75)	13	(21)	24	(8 to 40)	0.021	(-126 to 174)
*Prescribed medications*									
Cost (£)	44	69	(106)	97	(166)	-29	(-61 to 3)	0.702	(-234 to 177)

^a^Lin’s concordance correlation coefficient

## Discussion

### Main findings

ModRUM was piloted with 100 patients. Despite completion times being similar, based on response rates, ModRUM-C is potentially more acceptable to patients than ModRUM-CD. High question completion rates indicate that questions were feasible to answer. The results of this study provide some evidence for the construct and criterion validity of ModRUM for collecting resource-use data from patients recruited in a primary care setting. A revised version of ModRUM is available for use under license [30].

### Strengths and weaknesses of this study

This research presents initial testing of ModRUM. While testing of RUMs in trials prior to administration is not common [3], researchers will be encouraged to conduct their own testing prior to administration in a trial, to test ModRUM in their population and with any adaptations they have made. Due to Covid-19, patient recruitment could not be done face-to-face as planned. The sample was limited to 100 participants. Recruitment was instead conducted via postal invitation and the number of invitations sent was restricted by budgetary constraints. Acceptability was assessed through response rates and self-reported completion time. Response rates were higher for ModRUM-C than ModRUM-CD (20% versus 10%). These rates were consistent with previous research on response rates to postal surveys [31]. Response rates may have also been impeded by the Covid-19 pandemic. Invitations were sent during winter 2020/21, which included periods when England was in national lockdown, meaning people may have been less able or more reluctant to participate. In the context of a trial, for which ModRUM is designed to be used in, it is anticipated that response rates would be considerably higher given that trial participants are more likely to be engaged in the research. Question completion rates were at least 96% for ModRUM-C and 91% for ModRUM-CD.

Most participants completed the patient characteristics form and the EQ-5D-5L, which meant that construct validity could be tested. Support for the validity of ModRUM was obtained for hypotheses made regarding health-related quality of life and number of long-term conditions. Participants with lower EQ-5D-5L scores had higher total healthcare costs (*p* < 0.001). Participants with long-term conditions had higher total healthcare costs (*p* = 0.012). While it was hypothesised that older patients would have higher healthcare costs, the opposite was found with under 66-year-olds having higher costs (*p* = 0.002). It is likely that this result was impacted by the sampling strategy, where to be recruited to the study, patients were required to have had a recent appointment at their GP practice. This criterion means that the hypothesis, which was framed on the general population, may not be valid for this sample. Further testing of this hypothesis is required.

With the exception of one participant who had left their GP practice, medical record data was successfully obtained for all participants. Assessment of criterion validity was limited to a subset of ModRUM questions, where corresponding data were available in the primary care medical records. To assess the criterion validity of questions not assessed in this study, data would have needed to have been accessed from additional sources. Lower values of specificity may indicate that ModRUM is picking up resource use not captured in the medical records, or it could be a result of incorrectly reporting resource usage when it had not occurred within the recall period (telescoping). This included other primary and community-based healthcare professionals; however, these are unlikely to be comprehensively covered in the medical records, with services such as NHS 111 captured in ModRUM, but not in medical records. With good agreement observed for GP contacts and costs and prescribed medication costs, and results on average higher for GP and other health care professional contacts and costs in ModRUM, this study provides support for using ModRUM as an alternative to medical records for resource-use data. The increased proportion of contacts observed in ModRUM indicates that primary care medical records may not be a ‘gold standard’ for questions on primary and community care in ModRUM.

It was initially planned that patients would be recruited from GP practice waiting rooms; however, the Covid-19 pandemic meant that this was not feasible, so invitations and study documents were sent via the post, which limited the number of invitations that could be sent. Several strategies were adopted to maximise recruitment, such as invitations being addressed from patients’ GP practices. Although the response rate was comparable to previous postal surveys [31], other strategies to increase the response rate, such as reminders, may have increased the response rate further. However, medical record access restrictions meant that this would have needed to have been led by the GP practice, which would have added burden on the practices, and required additional funds.

As anticipated, practice deprivation level was associated with whether a patient participated. Increasing the number of invitations sent from practices in more deprived practices meant there was good representation from these practices. The sample included slightly more participants (55%) from the GP practices in the least deprived areas and more female participants (61%). Participants were mostly balanced across categories for number of long-term conditions and age on leaving full time education. The sample was predominantly of white ethnicity (95%), meaning non-white ethnic groups were not well represented in this pilot. Given the low proportion of people of non-white ethnicity at the participating GP practices, an alternative sampling approach may have helped to recruit people from non-white ethnic groups (e.g. a GP personally inviting patients to take part, and/or sending more invites to people from non-white ethnic groups). Equal representation was not achieved across age groups, with only one participant recruited from the 18 to 30 age group and 55% of participants aged 66 or over. This was expected based on the identification process, as patients were required to have had a clinical appointment at their GP practice in the last four weeks, and older patients were expected to visit the GP practice more frequently. Also, having a larger proportion of older participants may be more representative of the ages of people participating in many trials.

As recruitment in person was unfeasible, invitations were sent from GP practices via a hybrid mail service. GP practices uploaded patient details, and the mail service printed and sent the invitation packs. There were issues that may have impacted response rates and question completion rates for ModRUM-CD. First, due to the high-quality paper and the number of pages to return, the pre-paid envelope was almost too small. Several patients added notes to say the envelope was too small or they used their own envelope. Others may have completed the questionnaire, but not returned it. Second, the documentation was sent as individual sheets. A booklet may have improved question completion rates. For participants who had missed entire pages of ModRUM-CD, it was likely that the participant had not seen the question as opposed to missing it due to being unable to recall the data.

### Comparison to existing literature

To date, piloting of existing RUMs has been conducted to identify issues and refine RUMs, with the aim of improving acceptability to respondents and increasing data quality [27, 32–34]. Construct validity was assessed by Ness et al. for the Multiple Sclerosis Health Resource Utilization Survey [35]. All results were significant, including health-related quality of life, which was negatively associated with total costs, which is consistent with the result found in this study [35].

Many studies have compared self-report data with medical record data. Noben et al. reviewed studies reporting comparisons of self-report and administrative data and concluded that the evidence did not support one method over the other [36]. Of the six included studies that were considered to have sufficient quality, they concluded that patients generally reported lower estimates for resource use when compared with administrative data, which contrasts with the results of this study [36]. Patel et al. compared patient-report data from an adapted version of the client-service receipt inventory (CSRI) with GP medical record data for a random sample of primary care patients [37, 38]. They found no significant difference between data sources for number of contacts, with agreement, as estimated using Lin’s CCC, high (*p*_*c*_ = 0.756) [37]. More granular information was captured from participants for costing, which allowed self-report GP contacts to be costed by duration of consultation [37]. Byford et al. also used the CSRI and observed relatively high agreement for GP contacts (*p*_*c*_ = 0.631) [28]. A low level of agreement for other healthcare professionals in this study, was consistent with the findings of Byford et al., who found low levels of agreement for practice nurse, community psychologist and community psychiatric nurse contacts (all *p*_*c*_ < 0.350) [28]. Despite a difference in average cost, the good level of agreement of prescribed medication costs observed in this study (*p*_*c*_ = 0.702), was in contrast to existing research, where poor agreement has been observed [27].

### Implications for research practice

In this study, GP and other healthcare professional contacts were higher in ModRUM than GP medical records, suggesting that ModRUM may be more comprehensive for these resources. As healthcare providers become more diverse, for economic evaluation, a validated patient-report measure, which is relatively cheap to implement and easy to access, may be preferable to collecting medical record data from a range of sources. For administrative data to remain a feasible option for economic evaluations, increased diversity of providers means that data would ideally be obtained from a higher level (i.e. at integrated care system level, as opposed to individual providers).

For prescribed medications, high levels of sensitivity and specificity indicated that participant report was generally consistent with medical records for binary responses on whether participants had used medications. While the agreement between data sources was considered good, the total cost of prescribed medications was 42% higher when costed using medical record data. This could be the result of less accurate recall by participant report, assuming that it is unlikely that medications that were not prescribed would be included in medical records. The estimated cost difference could also have been impacted by the alternative costing approaches, with more detailed medical record data, allowing for increased precision in cost estimates.

Researchers should carefully consider the amount of detail they ask participants to provide. Both the response rate and question completion rates were higher for the shorter version of ModRUM. Where researchers are uncertain what level of detail is appropriate to collect, depth questions could be included in the feasibility or internal pilot phase of a randomised controlled trial. Costing this information using both top-level core questions and detailed information from tables, could indicate whether questions can be made more concise. If the researcher chooses to use core questions in the main trial, the detail provided during the internal pilot or feasibility study could also be used to inform the most appropriate unit costs to use in the final analysis. For example, if a large proportion of outpatient appointments are performed in Orthopaedics, the researcher may choose an orthopaedics unit cost to cost all appointments, as opposed to a generic outpatient unit cost.

### Unanswered questions and future research

Further testing of ModRUM is required in a larger sample, in trials and with groups that were not well represented in this study (non-white ethnic groups and people aged 30 and under). Further research is underway to increase the breadth of ModRUM, with modules covering social care and informal care. This research reports the development of a paper-based version of ModRUM, an electronic version is also being developed.

## Conclusion

This study provides preliminary evidence for the feasibility, acceptability, and construct and criterion validity of ModRUM in a sample of patients recruited from primary care. Future testing of ModRUM is required within trials, and with groups that were less well-represented in this study.

### Supplementary Information

**Additional file 1: Figure S1.** ModRUM core module with depth questions. **Table S1.** Unit costs, by healthcare resource. **Table S2.** Participant-reported time to complete ModRUM. **Table S3.** Participant characteristics. **Table S4.** EQ-5D-5L scores, by ModRUM version. **Table S5.** Healthcare utilisation and costs, by ModRUM version. **Table S6.** Comparison of costs for participants who completed ModRUM-CD, using information from core and depth questions

## Data Availability

The datasets used and analysed during the current study are available from the corresponding author upon reasonable request.

## Abbreviations

CCC: Concordance Correlation Coefficient
CSRI: Client Service Receipt Inventory
GP: General practitioner
ModRUM: Modular resource-use measure
ModRUM-C: ModRUM core module
ModRUM-CD: ModRUM core module with depth questions
RUM: Resource-use measure
