# Supplementary material for: Development of a brief, generic, modular resource-use measure (ModRUM): piloting with patients
Source: BMC Health Serv Res. 2023 Sep 15;23:994. doi: 10.1186/s12913-023-10011-x (PMC10503201; doi:10.1186/s12913-023-10011-x)
Supplement: Supplementary file 1 — Additional file 1: Figure S1. ModRUM core module with depth questions. Table S1. Unit costs, by healthcare resource. Table S2. Participant-reported time to complete ModRUM. Table S3. Participant characteristics. Table S4. EQ-5D-5L scores, by ModRUM version. Table S5. Healthcare utilisation and costs, by ModRUM version. Table S6. Comparison of costs for participants who completed ModRUM-CD, using information from core and depth questions [file 12913_2023_10011_MOESM1_ESM.pdf]

ADDITIONAL FILE 1

Figure S1. ModRUM core module with depth questions

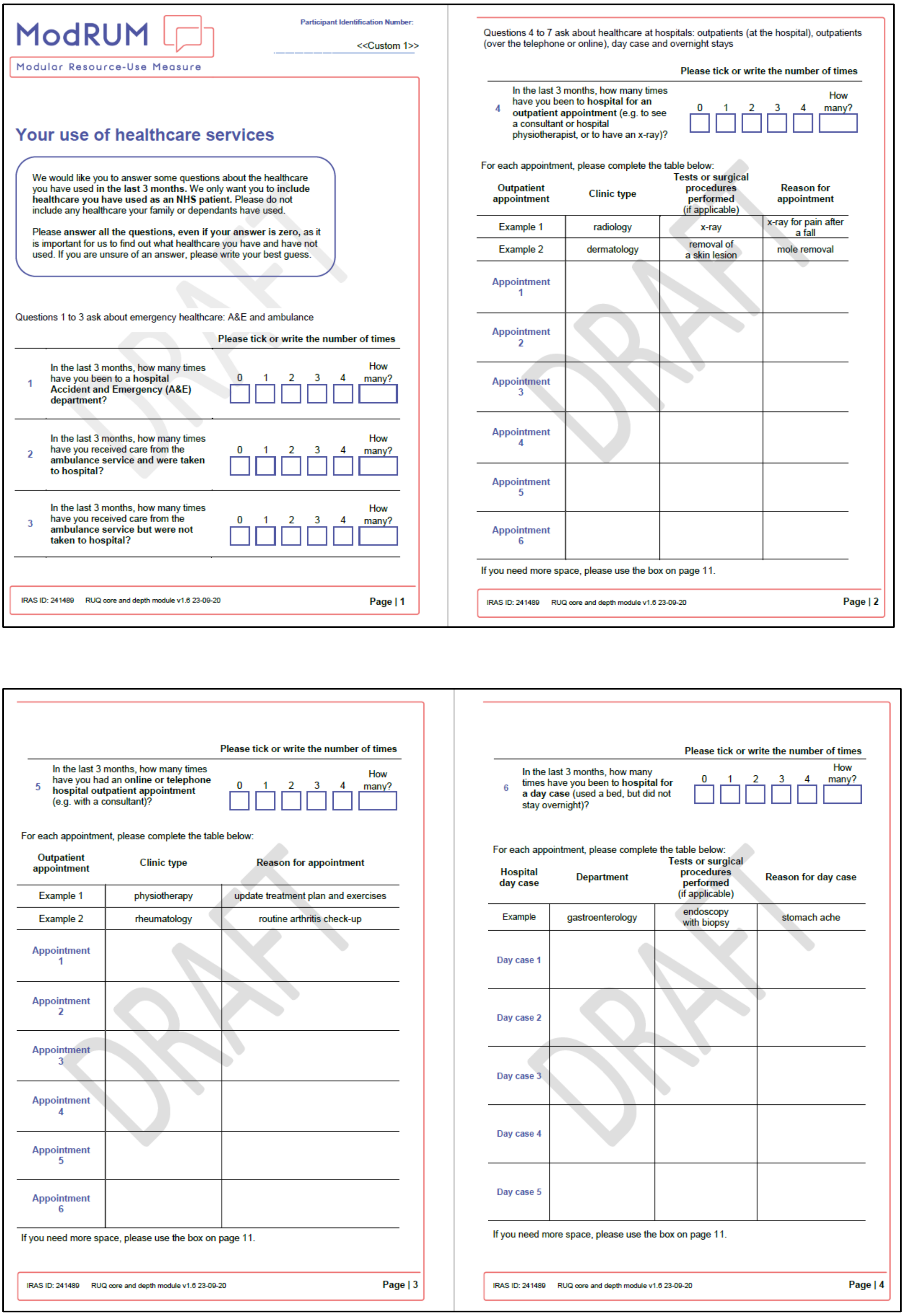

ModRUM
Modular Resource-Use Measure

Participant Identification Number:
<<Custom 1>>

Your use of healthcare services

We would like you to answer some questions about the healthcare you have used **in the last 3 months.** We only want you to **include healthcare you have used as an NHS patient.** Please do not include any healthcare your family or dependants have used.

Please **answer all the questions, even if your answer is zero,** as it is important for us to find out what healthcare you have and have not used. If you are unsure of an answer, please write your best guess.

DRAFT

Questions 1 to 3 ask about emergency healthcare: A&E and ambulance

		Please tick or write the number of times
1	In the last 3 months, how many times have you been to a hospital **Accident and Emergency (A&E) department?**	0 ☐ 1 ☐ 2 ☐ 3 ☐ 4 ☐ How many? ☐
2	In the last 3 months, how many times have you received care from the **ambulance service and were taken to hospital?**	0 ☐ 1 ☐ 2 ☐ 3 ☐ 4 ☐ How many? ☐
3	In the last 3 months, how many times have you received care from the **ambulance service but were not taken to hospital?**	0 ☐ 1 ☐ 2 ☐ 3 ☐ 4 ☐ How many? ☐

IRAS ID: 241489 RUQ core and depth module v1.6 23-09-20 **Page | 1**

Questions 4 to 7 ask about healthcare at hospitals: outpatients (at the hospital), outpatients (over the telephone or online), day case and overnight stays

		Please tick or write the number of times
4	In the last 3 months, how many times have you been to **hospital for an outpatient appointment** (e.g. to see a consultant or hospital physiotherapist, or to have an x-ray)?	0 ☐ 1 ☐ 2 ☐ 3 ☐ 4 ☐ How many? ☐

For each appointment, please complete the table below:

Outpatient appointment	Clinic type	Tests or surgical procedures performed (if applicable)	Reason for appointment
Example 1	radiology	x-ray	x-ray for pain after a fall
Example 2	dermatology	removal of a skin lesion	mole removal
Appointment 1			
Appointment 2			
Appointment 3			
Appointment 4			
Appointment 5			
Appointment 6			

If you need more space, please use the box on page 11.

DRAFT

IRAS ID: 241489 RUQ core and depth module v1.6 23-09-20 **Page | 2**

		Please tick or write the number of times
5	In the last 3 months, how many times have you had an **online or telephone hospital outpatient appointment** (e.g. with a consultant)?	0 ☐ 1 ☐ 2 ☐ 3 ☐ 4 ☐ How many? ☐

For each appointment, please complete the table below:

Outpatient appointment	Clinic type	Reason for appointment
Example 1	physiotherapy	update treatment plan and exercises
Example 2	rheumatology	routine arthritis check-up
Appointment 1		
Appointment 2		
Appointment 3		
Appointment 4		
Appointment 5		
Appointment 6		

If you need more space, please use the box on page 11.

DRAFT

IRAS ID: 241489 RUQ core and depth module v1.6 23-09-20 **Page | 3**

		Please tick or write the number of times
6	In the last 3 months, how many times have you been to **hospital for a day case** (used a bed, but did not stay overnight)?	0 ☐ 1 ☐ 2 ☐ 3 ☐ 4 ☐ How many? ☐

For each appointment, please complete the table below:

Hospital day case	Department	Tests or surgical procedures performed (if applicable)	Reason for day case
Example	gastroenterology	endoscopy with biopsy	stomach ache
Day case 1			
Day case 2			
Day case 3			
Day case 4			
Day case 5			

If you need more space, please use the box on page 11.

DRAFT

IRAS ID: 241489 RUQ core and depth module v1.6 23-09-20 **Page | 4**

Figure S1 continued

Please tick or write the number of times

		0	1	2	3	4	How many?
7	In the last 3 months, how many times have you been to **hospital for an overnight stay**?	☐	☐	☐	☐	☐	☐

For each appointment, please complete the table below:

Hospital overnight stay	Number of nights spent in hospital	Department	Tests or surgical procedures performed (if applicable)	Reason for stay
Example	4	orthopaedics	knee replacement	knee replacement
Stay 1				
Stay 2				
Stay 3				
Stay 4				
Stay 5				

If you need more space, please use the box on page 11.

DRAFT

Questions 8 to 16 ask about healthcare outside of hospitals: GP (doctor), nurse and any other NHS healthcare professionals or services

Please tick or write the number of times

		0	1	2	3	4	How many?
8	In the last 3 months, how many times have you had an appointment with a **doctor (GP) at a GP surgery, health centre or walk-in centre?**	☐	☐	☐	☐	☐	☐
9	In the last 3 months, how many times have you had an appointment with a **doctor (GP) over the telephone or online** ?	☐	☐	☐	☐	☐	☐
10	In the last 3 months, how many times have you had an appointment with a **doctor (GP) at home?**	☐	☐	☐	☐	☐	☐
11	In the last 3 months, how many times have you had an appointment/contact with **a nurse at a GP surgery, health centre or walk-in centre?**	☐	☐	☐	☐	☐	☐
12	In the last 3 months, how many times have you had an appointment/contact with **a nurse** over **the telephone or online?**	☐	☐	☐	☐	☐	☐
13	In the last 3 months, how many times have you had an appointment/contact with **a nurse** at **home?**	☐	☐	☐	☐	☐	☐

DRAFT

Please tick or write the number of times

		0	1	2	3	4	How many?
14	In the last 3 months, how many times have you had contact with **any other NHS healthcare professionals** (e.g. community physiotherapist) **or NHS healthcare services at a GP surgery, health centre or walk-in centre?**	☐	☐	☐	☐	☐	☐

For each healthcare professional seen and/or healthcare service you have used, please complete the table below:

Healthcare professional or service	Healthcare professional seen or healthcare service used	Number of times
Example	community physiotherapist	3
Healthcare professional or service 1		
Healthcare professional or service 2		
Healthcare professional or service 3		
Healthcare professional or service 4		
Healthcare professional or service 5		

If you need more space, please use the box on page 11.

DRAFT

Please tick or write the number of times

		0	1	2	3	4	How many?
15	In the last 3 months, how many times have you had contact with **any other NHS healthcare professionals or NHS healthcare services** (e.g. NHS 111 telephone call) over the **telephone or online?**	☐	☐	☐	☐	☐	☐

For each healthcare professional seen and/or healthcare service you have used, please complete the table below:

Healthcare professional or service	Healthcare professional seen or healthcare service used	Number of times
Example	NHS 111 telephone call	1
Healthcare professional or service 1		
Healthcare professional or service 2		
Healthcare professional or service 3		
Healthcare professional or service 4		
Healthcare professional or service 5		

If you need more space, please use the box on page 11.

DRAFT

Figure S1 continued

Please tick or write the number of times

16 In the last 3 months, how many times have you had contact with any other **NHS healthcare professionals** (e.g. health visitor) or **NHS healthcare services at home?**

0	1	2	3	4	How many?
☐	☐	☐	☐	☐	☐

For each healthcare professional seen and/or healthcare service you have used, please complete the table below:

Healthcare professional or service	Healthcare professional seen or healthcare service used	Number of times
Example	NHS chiropodist	3
Healthcare professional or service 1		
Healthcare professional or service 2		
Healthcare professional or service 3		
Healthcare professional or service 4		
Healthcare professional or service 5		

If you need more space, please use the box on page 11.

DRAFT

Question 17 asks about prescribed medications

Please tick

17 In the last 3 months, have you picked up or received any **prescribed medications?**

yes	no
☐	☐

For each prescribed medication, please complete the table:

Prescribed medication	Name of prescribed medication<br>If you don't know the name, please put the type of medication	Number of times the medication has been picked up and/or received
Example 1	Tramadol / Painkiller	3
Example 2	Diprobase / Eczema cream	1
Medication 1		
Medication 2		
Medication 3		
Medication 4		
Medication 5		
Medication 6		
Medication 7		
Medication 8		
Medication 9		
Medication 10		

If you need more space, please use the box on page 11.

DRAFT

If you run out of space in the tables, please use the box below to write about the other healthcare you have used:

DRAFT

Please check you have answered every question.

Thank you for completing the questionnaire.

How long did it take you to fill in this questionnaire?
.... **minutes**

Table S1. Unit costs, by healthcare resource

Healthcare resource	Unit cost (£)	Source(s)
Ambulance (see and treat or refer)	209.38	(1)
Ambulance (see and treat and convey)	257.34	(1)
A&E	166.05	(1)
Outpatient appointment (face-to-face)	124.81[1]	(1)
Outpatient appointment (non-face-to-face)	77.89[1]	(1)
Day case	755.59[1]	(1)
Inpatient stay (per night)	535.41[1]	(1, 2)
GP (surgery/clinic)	33.19	(3)
GP (home)	84.24	(3, 4)
GP (online/telephone)	25.56	(3, 4)
Nurse (surgery/clinic)	16.28	(3, 5)
Nurse (home)	39.68	(1)
Nurse (online/telephone)	6.30	(3, 5)
OHCP[2] (surgery/clinic)	16.28[1]	(3, 5)
OHCP[2] (home)	39.68[1]	(1)
OHCP[2] (online/telephone)	18.28[1]	(1, 3, 6)
Prescribed medications	Varies[3]	(7)

[1]This is an average unit cost, used for costing core questions, for participants who provided more information in ModRUM-CD (e.g. tests/procedures performed for outpatients or physiotherapist for oHCP seen), the unit cost varies, [2]Other healthcare professional, [3]Costed using name and dose (where provided) using cost per item

Table S2. Participant-reported time to complete ModRUM

	n	Mean minutes (SD)		Median minutes (IQR)		[Min, Max]
Core	42	5.4	(4.3)	5.0	(3.0-6.0)	[1, 25]
Core with depth	40	5.7	(3.8)	5.0	(2.5-8.0)	[1, 12]

Table S3. Participant characteristics

		n	(%)
Female		61	(61)
White ethnicity		95	(95)
Age group	18-30	1	(1)
	31-45	14	(14)
	46-55	15	(15)
	56-65	15	(15)
	66-75	32	(32)
	76 or over	23	(23)
Long term conditions	More than one	34	(34)
	One	24	(24)
	None	39	(39)
Age on leaving full-time education	16 or under	44	(44)
	17 or 18	18	(18)
	19 or over	35	(35)
Practice deprivation score*	2	10	(10)
	4	18	(18)
	5	17	(17)
	10	55	(18)

*1=most deprived, 10=least deprived

Table S4. EQ-5D-5L scores, by ModRUM version

	n	(%)	Mean	(SD)	[Min, Max]
**All (N=100)**					
Utility score	99	(99)	0.750	(0.249)	[-0.227, 1]
VAS score	96	(96)	76	(20)	[0, 100]
**Core (N=53)**					
Utility score	52	(98)	0.772	(0.212)	[-0.200, 1]
VAS score	51	(96)	77	(20)	[0, 100]
**Core plus depth (N=47)**					
Utility score	47	(100)	0.726	(0.285)	[-0.227, 1]
VAS score	45	(96)	75	(21)	[0, 100]

VAS: Visual analogue scale

Table S5. Healthcare utilisation and costs, by ModRUM version

	Core module (N=53)						Core module with depth questions (N=47)					
			Resource use		Cost (£)				Resource use		Cost (£)	
	n	(%)	Mean	[Min, Max]	Mean	(SD)	n	(%)	Mean	[Min, Max]	Mean	(SD)
A&E	53	(100)	0.15	[0, 1]	25	(60)	47	(100)	0.17	[0, 2]	28	(72)
Ambulance (convey)	-	-	-	-	-	-	47	(100)	0.06	[0, 1]	13	(52)
Ambulance (treat)	-	-	-	-	-	-	47	(100)	0.04	[0, 1]	11	(53)
Outpatient (f2f)	53	(100)	0.58	[0, 6]	72	(132)	46	(98)	0.61	[0, 5]	78	(158)
Outpatient (remote)	53	(100)	0.36	[0, 3]	28	(52)	46	(98)	0.43	[0, 4]	35	(71)
Day case	53	(100)	0.17	[0, 4]	128	(486)	46	(98)	0.07	[0, 1]	72	(322)
Inpatient stays	53	(100)	0.08	[0, 2]	101	(493)	47	(100)	0.06	[0, 1]	281	(1162)
GP (surgery)	52	(98)	0.60	[0, 4]	20	(29)	45	(96)	1.18	[0, 7]	39	(41)
GP (remote)	52	(98)	1.90	[0, 10]	49	(42)	44	(94)	1.84	[0, 12]	47	(48)
GP (home)	52	(98)	0.08	[0, 4]	6	(47)	44	(94)	0.00	[0, 0]	0	(0)
Nurse (surgery)	-	-	-	-	-	-	45	(96)	0.84	[0, 5]	8	(10)
Nurse (remote)	-	-	-	-	-	-	44	(94)	0.20	[0, 2]	1	(2)
Nurse (home)	-	-	-	-	-	-	44	(94)	0.41	[0, 14]	16	(84)
Other HCP (surgery)	52	(98)	1.00	[0, 7]	16	(27)	47	(100)	0.28	[0, 4]	14	(51)
Other HCP (remote)	52	(98)	0.33	[0, 2]	6	(11)	43	(91)	0.35	[0, 5]	8	(28)
Other HCP (home)	52	(98)	0.15	[0, 7]	6	(39)	46	(98)	0.09	[0, 2]	7	(32)
Medications	-	-	-	-	-	-	45	(96)	-	-	67	(105)
**Mean total cost**	52	(98)	-	-	462	(802)	38	(81)	-	-	537	(1045)

*HCP: healthcare professional

Table S6. Comparison of costs for participants who completed ModRUM-CD, using information from core and depth questions

	n	Mean resource use	ModRUM-CD costs (£)		ModRUM-C costs (£)	
			Mean	(SD)	Mean	(SD)
Outpatient (f2f)	46	0.61	78	(158)	75	(139)
Outpatient (remote)	46	0.43	35	(71)	33	(66)
Day case	46	0.07	72	(322)	49	(189)
Inpatient stays	47	0.06	281	(1162)	228	(1132)
Other HCP (surgery)[12]	45	1.04	17	(36)	10	(13)
Other HCP (remote)[12]	41	0.59	9	(28)	11	(10)
Other HCP (home)[12]	43	0.51	24	(94)	20	(88)
Total	39		347	(956)	246	(550)

1: HCP=healthcare professional; 2: Depth=nurse plus other health care professional

1. NHS England and NHS Improvement. National Schedule of NHS Costs 2018/19. 2020 [Available from: https://www.england.nhs.uk/national-cost-collection/].
2. NHS Digital. Mean and median length of stay of hospital inpatient episodes 2019 [Available from: https://digital.nhs.uk/data-and-information/find-data-and-publications/supplementary-information/2019-supplementary-information-files/mean-and-median-length-of-stay-of-hospital-inpatient-episodes].
3. Curtis LA, Burns A. Unit Costs of Health & Social Care 2020. University of Kent: PSSRU; 2020.
4. Curtis LA. Unit Costs of Health & Social Care 2013. University of Kent: PSSRU; 2013.
5. Curtis LA, Burns A. Unit Costs of Health & Social Care 2015. University of Kent: PSSRU; 2015.
6. Turner J, O'Cathain A, Knowles E, Nicholl J, Tosh J, Sampson F, et al. Evaluation of NHS 111 pilot sites 2012 [Available from: https://www.sheffield.ac.uk/polopoly_fs/1.227404%21/file/NHS_111_final_report_August_2012.pdf].
7. NHS Business Services Authority. Prescription Cost Analysis - England 2019. 2020.
